# Using CRISPR-Cas9/phosphoproteomics to identify substrates of calcium/calmodulin-dependent kinase 2δ

**DOI:** 10.1016/j.jbc.2023.105371

**Published:** 2023-10-20

**Authors:** Euijung Park, Chin-Rang Yang, Viswanathan Raghuram, Lihe Chen, Chung-Lin Chou, Mark A. Knepper

**Affiliations:** Epithelial Systems Biology Laboratory, Systems Biology Center, National Heart, Lung, and Blood Institute, NIH, Bethesda, Maryland, USA

**Keywords:** aquaporin-2, vasopressin, mass spectrometry

## Abstract

Ca^2+^/Calmodulin-dependent protein kinase 2 (CAMK2) family proteins are involved in the regulation of cellular processes in a variety of tissues including brain, heart, liver, and kidney. One member, CAMK2δ (CAMK2D), has been proposed to be involved in vasopressin signaling in the renal collecting duct, which controls water excretion through regulation of the water channel aquaporin-2 (AQP2). To identify CAMK2D target proteins in renal collecting duct cells (mpkCCD), we deleted *Camk2d* and carried out LC-MS/MS–based quantitative phosphoproteomics. Specifically, we used CRISPR/Cas9 with two different guide RNAs targeting the CAMK2D catalytic domain to create multiple CAMK2D KO cell lines. AQP2 protein abundance was lower in the CAMK2D KO cells than in CAMK2D-intact controls. AQP2 phosphorylation at Ser256 and Ser269 (normalized for total AQP2) was decreased. However, trafficking of AQP2 to and from the apical plasma membrane was sustained. Large-scale quantitative phosphoproteomic analysis (TMT-labeling) in the presence of the vasopressin analog dDAVP (0.1 nM, 30 min) allowed quantification of 11,570 phosphosites of which 169 were significantly decreased, while 206 were increased in abundance in CAMK2D KO clones. These data are available for browsing or download at https://esbl.nhlbi.nih.gov/Databases/CAMK2D-proteome/. Motif analysis of the decreased phosphorylation sites revealed a target preference of -(R/K)-X-X-p(S/T)-X-(D/E), matching the motif identified in previous *in vitro* phosphorylation studies using recombinant CAMK2D. Thirty five of the significantly downregulated phosphorylation sites in CAMK2D KO cells had exactly this motif and are judged to be likely direct CAMK2D targets. This adds to the list of known CAMK2D target proteins found in prior reductionist studies.

Changes in intracellular calcium concentration are integral to many signaling pathways. Signaling through calcium mobilization occurs in part through Ca^2+^/calmodulin-dependent effectors including many protein kinases. These effectors include Ca^2+^/calmodulin-dependent protein kinases (CAMK) 2α, 2β, 2γ, and 2δ (1, 2). CAMK2α and 2β are mainly expressed in brain, while 2δ and 2γ are broadly distributed among tissues (https://gtexportal.org/home/). CAMK2 family kinases are involved in the regulation of cellular processes in a variety of tissues including brain, heart, liver, and kidney (3, 4). RNA-seq analysis of microdissected mouse kidney tubules and the mouse cortical collecting duct mpkCCD cell line identified the expression of four Camk2 isoforms (protein name: CAMK2, gene symbol in mouse: *Camk2a*, *Camk2b*, *Camk2d*, and *Camk2g*) (https://esbl.nhlbi.nih.gov/MRECA/Nephron/) (5, 6), with the *Camk2d* being the most abundant transcript in collecting duct. The abundance of the *Camk2d* transcript seems to be unaffected by vasopressin (6). Quantitative proteomic analysis of rat kidney tubules also showed that CAMK2D and CAMK2G isoforms are expressed in all renal tubule segments, with CAMK2D being the dominant form (7). It is particularly strongly expressed in collecting duct principal cells where it has been proposed to be involved in vasopressin signaling (4, 8). In isolated perfused collecting ducts, calmodulin inhibitors and calcium chelators have been shown to attenuate vasopressin’s effect to increase osmotic water transport (9, 10, 11).

Vasopressin (arginine vasopressin, AVP) is a peptide hormone secreted by the posterior pituitary gland in response to high blood osmolality, usually due to systemic water deficits (dehydration). It controls water excretion in part through regulation of the water channel aquaporin-2 (AQP2) both through regulated trafficking of AQP2 to the plasma membrane and through regulated transcription of the *Aqp2* gene (12, 13, 14, 15, 16). Vasopressin acts by binding to the vasopressin V2 receptor, which signals *via* the heterotrimeric G-protein α subunit G_αs_, resulting in the activation of adenylyl cyclase 6 and cAMP-mediated activation of the protein kinase A (PKA) (17, 18). Downstream from PKA is a broad signaling network, associated with secondary activation of multiple protein kinases (19), rearrangement of the actin cytoskeleton (9, 20, 21, 22, 23), and intracellular calcium mobilization (10, 24, 25). The latter is thought to occur in part through PKA-mediated phosphorylation of the endoplasmic reticulum calcium channel Itpr3 (18, 26). The resulting calcium mobilization occurs as a series of calcium spikes in collecting duct cells with its frequency increased when vasopressin is present (10, 27). Calcium mobilization then is believed to activate calmodulin-dependent kinases including myosin light chain kinase (MLCK) (9) and CAMK2D (8). In previous studies, we identified phosphorylation targets of MLCK by deleting the *Mylk* gene using CRISPR-Cas9 followed by phosphoproteomic quantification of all phosphorylation sites whose phospho-occupancies are affected by the gene knockout (28).

The objective of this paper is to identify downstream targets of CAMK2D using CRISPR-Cas9 to delete *Camk2d* and then observe changes in the phosphoproteome in mpkCCD cells. These cells have been shown to exhibit features of vasopressin-mediated regulation of AQP2 including increased membrane trafficking of AQP2 protein to the apical plasma membrane (29) and increased transcription of the *Aqp2* gene (30).

## Results

### CRISPR-Cas9 deletion of *Camk2d* gene

**Figure 1:**
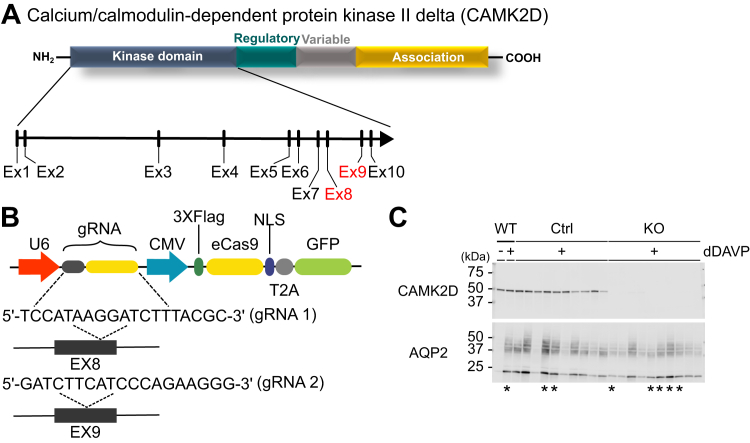
**Generation of calcium/calmodulin-dependent kinase II δ KO mpkCCD cells.***A*, exons 8 and 9 which code for the catalytic domain of Camk2d were targeted by two separate gRNAs. *B*, actual sequences of the two gRNAs. *C*, semiquantitative immunoblots show responses to long-term dDAVP exposure. Clones that underwent the transfection procedure but lacked mutations in *Camk2d* were used as *Camk2d*-intact cells. *Camk2d*-KO clones maintained expression of AQP2 protein in the presence of dDAVP. Clones which are marked with ∗ below their respective immunoblot lanes were selected for the further experiments. AQP2, aquaporin-2; CAMK2D, CAMK2δ; dDAVP, 1-desamino-8-D-arginine-vasopressin; gRNA, guide RNA.

We used mpkCCDc11-38 cells (28), a cell line that manifests vasopressin-mediated AQP2 trafficking and regulation of transcription of the *Aqp2* gene, to generate *Camk2d*-KO cells. Specifically, we used CRISPR-Cas9 to introduce mutations that ablate *Camk2d* gene expression. For this, we used guide RNAs (gRNAs) targeting two different sites corresponding to exons 8 and 9 (independently transfected), which code for the kinase domain (catalytic region) of the CAMK2D protein (Fig. 1, *A* and *B*). Single-cell–derived clones with successful *Camk2d* deletion (KO) were identified by immunoblotting (Fig. 1*C*). These are considered biological replicates in subsequent experiments. Clones that underwent the transfection procedure but retained CAMK2D expression were chosen as Control (Ctrl) clones (considered biological replicates). We retained the parental line as an additional control (additional biological replicate). AQP2 abundance in the presence of the V2R selective-vasopressin analog 1-desamino-8-D-arginine-vasopressin (dDAVP) (0.1 nM) was variable among the clones and was not clearly ablated in CAMK2D KO cells. Clones selected for further analysis are indicated by asterisks (Fig. 1*C*). Mutations were confirmed by Sanger sequencing (Table S1).

### Characterization of *Camk2d* KO cells

**Figure 2:**
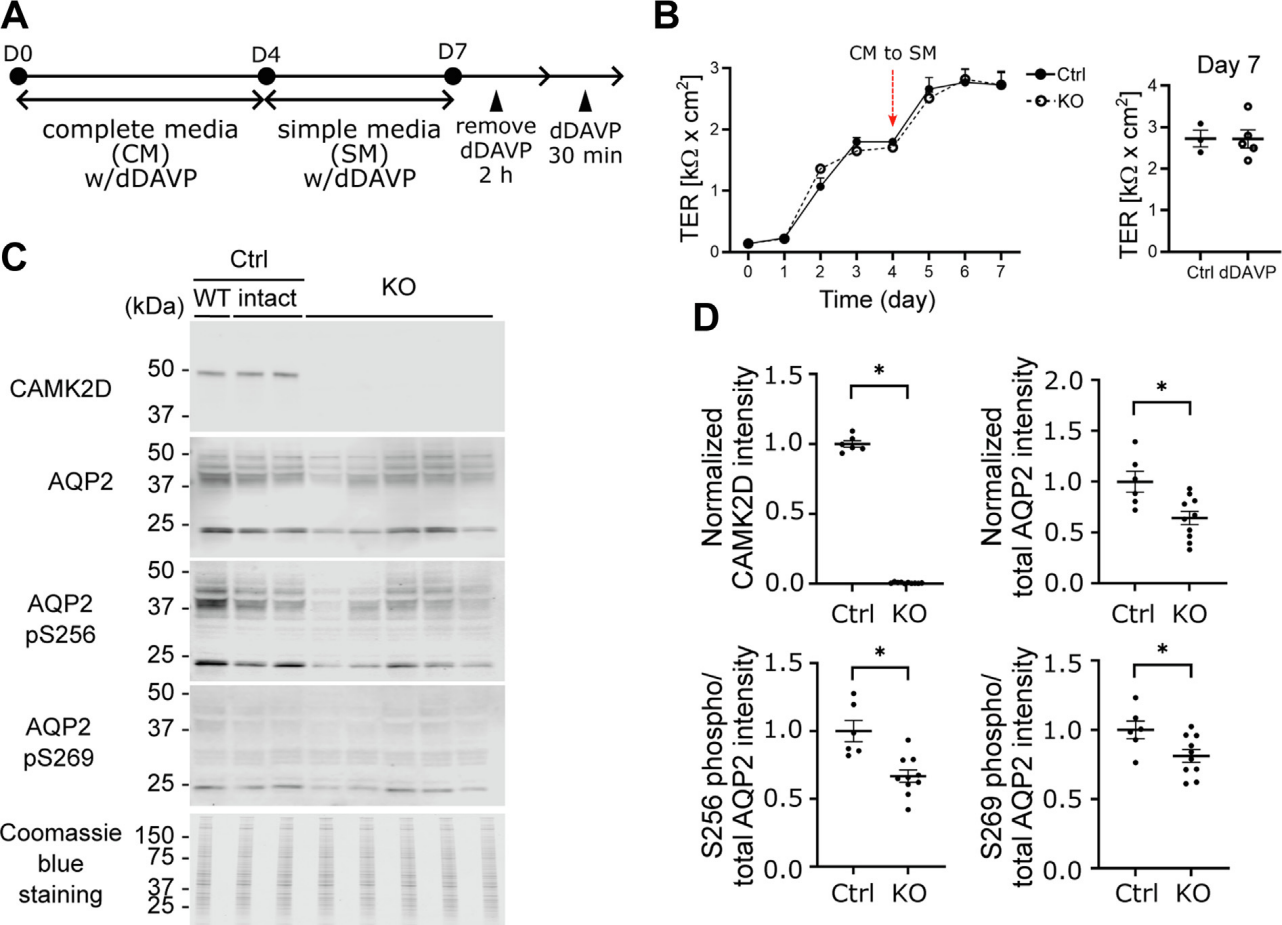
**Phosphorylation of AQP2 in *Camk2d* KO cells.***A*, cell-culture strategy for short-term dDAVP (30 min, 0.1 nM) treatment. *B*, development of transepithelial resistance (TER). Cells developed high transepithelial resistance as a function of time but did not show differences between *Camk2d*-intact and *Camk2d*-KO cells. *C* and *D*, semiquantitative immunoblots and densitometry of CAMK2D, total AQP2, phosphorylated AQP2 (at Ser256 and Ser269) in response to dDAVP. The abundance of total AQP2 and ratios of pS256 and pS269 to total AQP2 were significantly decreased in *Camk2d*-KO clones compared to *Camk2d* intact clones. ∗*p* < 0.05. unpaired *t* test. AQP2, aquaporin-2; CAMK2D, CAMK2δ; dDAVP, 1-desamino-8-D-arginine-vasopressin.

**Figure 3:**
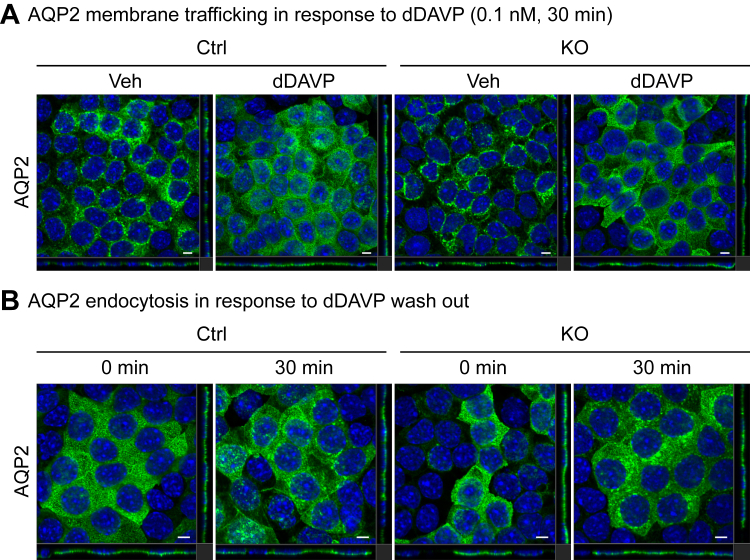
**Subcellular distribution of AQP2 in *Camk2d* KO cells.***A*, representative confocal images of *Camk2d*-intact and *Camk2d*-KO cells labeled with anti-AQP2 antibody in the presence of 0.1 nM dDAVP for 30 min (*green*). Apical distribution of AQP2 was detected both in *Camk2d*-intact and KO cells in response to dDAVP stimulation. *B*, representative confocal images of *Camk2d* intact and KO cells labeled with anti-AQP2 antibody with dDAVP washout for 30 min (*green*). Internalization of AQP2 was detected both in *Camk2d* intact and KO cells after dDAVP washout showing typical punctate labeling in cytoplasm. Confocal microscope magnification 63×. Scale bar represents 5 μm. DAPI labeling indicated in *blue*. AQP2, aquaporin-2; CAMK2D, CAMK2δ; dDAVP, 1-desamino-8-D-arginine-vasopressin.

**Figure 4:**
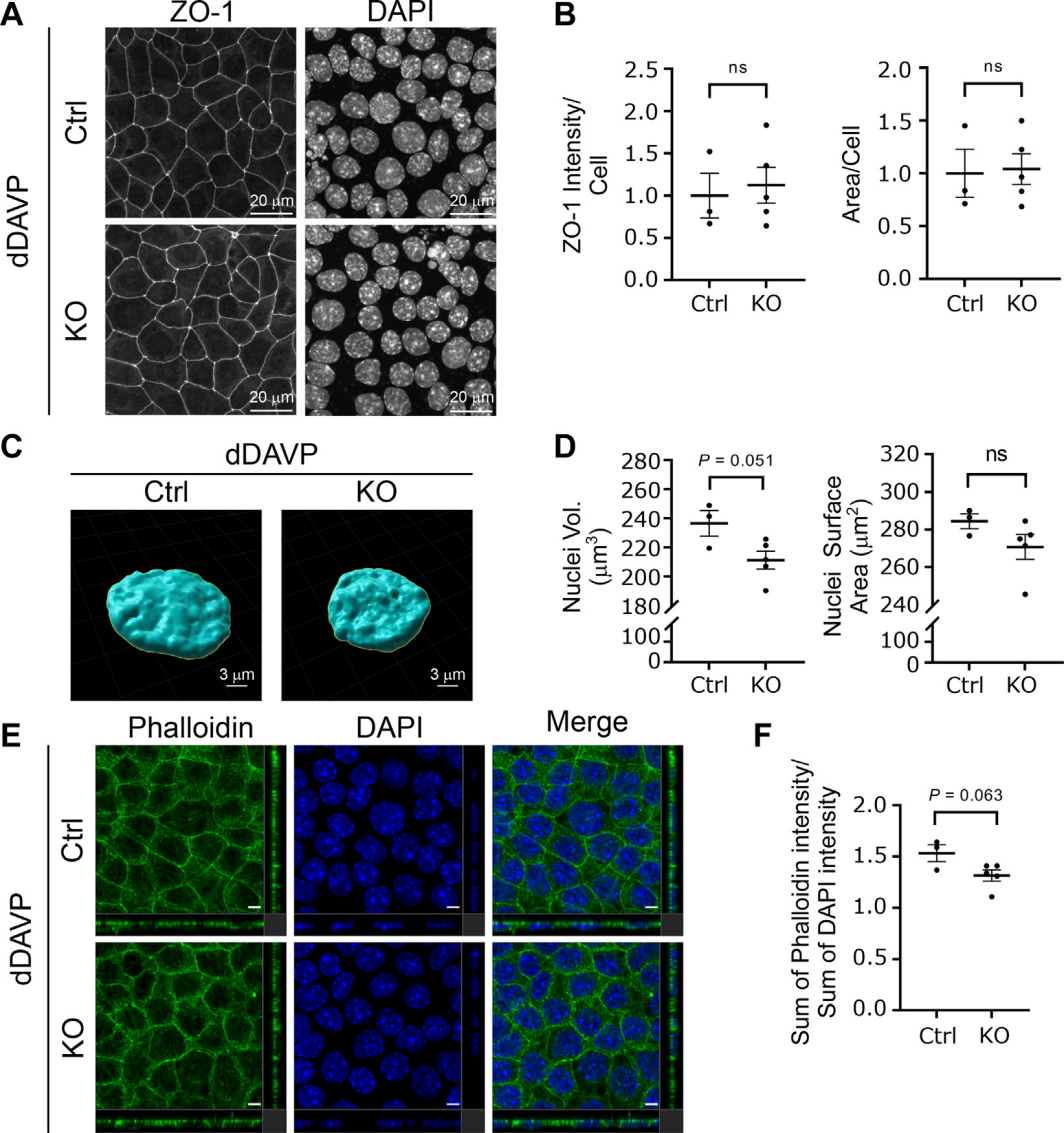
**Tight junction, distribution of actin cytoskeleton, and nuclear morphology in the presence of dDAVP.***A* and *B*, confocal images of *Camk2d* intact and KO cells labeled with antibody recognizing Zonula Occludens 1 (ZO-1) protein. Epithelial polarization and the cell cross sectional area was unaffected by *Camk2d* deletion. The ZO-1 intensity and cross sectional area were calculated from the cell shown entire image field using ImageJ. Nuclear counts per unit area were used to estimate cell density. Maximum intensity Z-projection of ZO-1 was divided by the number of cells. Three randomly chosen areas from three intact and five KO cell lines were used for analysis. *C* and *D*, nuclei volume and surface areas in intact and KO cells. Nuclei volume and nuclear surface area were calculated with Imaris software. The calculation showed no significant changes in *Camk2d* KO cells compared to the intact cells. Nuclei volume and surface area were calculated using the *Surfaces* tool of Imaris. A total of 240 nuclei from intact cells (three random area per clone, three clones) and total 468 nuclei from KO cells (three random area per clone, five clones) were analyzed. *E* and *F*, confocal images of *Camk2d* intact and KO cells labeled with phalloidin. Subcellular distribution of F-actin was not demonstrably changed with *Camk2d* deletion. The sum of phalloidin intensities and the sum of DAPI intensities were obtained from Imaris. The nuclei were stained by 4,6-diamidino-2-phenylindole (DAPI). Morphometry was carried out in three randomly selected areas for both intact and KO clones. Confocal microscope magnification 63×. Scale bar without a number represents 5 μm. Three randomly chosen areas from three intact and five KO cell lines were used for analysis. *p* values are from unpaired t-tests throughout. CAMK2D, CAMK2δ; dDAVP, 1-desamino-8-D-arginine-vasopressin.

Both control (Ctrl) and KO clones were grown on membrane filters and treated with dDAVP as described in Figure 2*A*. The KO cells grew at a rate similar to the control cells and were morphologically indistinguishable during the culture period. Both *Camk2d* KO and Ctrl clones started forming polarized epithelia in 2 days as indicated by increasing transepithelial electrical resistance, which reached a maximum level in the experimental day 7 (Fig. 2*B*). Transepithelial electrical resistance was not affected by *Camk2d* deletion (Fig. 2*B*). Semiquantitative immunoblotting showed absence of CAMK2D protein (Fig. 2, *C* and *D*). The abundance of total AQP2 was lower in *Camk2d* KO clones (KO/Ctrl = 0.36 ± 0.12, *p* < 0.05) (Fig. 2, *C* and *D*). Also, phosphorylation of AQP2 at serine 256 and 269 were attenuated in KO clones (phosphorylated/total KO/Ctrl = 0.22 ± 0.08 and 0.18 ± 0.08, *p* < 0.05, respectively) (Fig. 2, *C* and *D*). AQP2 trafficking to the apical membrane was examined by immunofluorescence (Fig. 3*A*). In both control and KO cells in the absence of dDAVP, AQP2 is distributed largely within intracellular vesicles. dDAVP caused redistribution of AQP2 to the apical region of the cells in both control and KO cells, best seen in the XZ and YZ cross sections. Therefore, *Camk2d* deletion did not prevent dDAVP-induced redistribution of AQP2 to the apical region (Fig. 3*A*). To examine the endocytosis of AQP2 from the apical plasma membrane, dDAVP was removed from the cells for 30 min, and immunofluorescence staining was carried out with an AQP2 antibody (Fig. 3*B*). In *Camk2d*-intact cells, the redistribution of AQP2 was observed with dDAVP washout, showing the typical punctate pattern indicative of internalization into endosomes (Fig. 3*B*). There was no obvious difference between intact and KO cells. The observation indicated that the *Camk2d* KO did not substantially affect the ability of the cells to endocytose AQP2 (Fig. 3*B*). ZO-1 immunofluorescence staining (Fig. 4*A*) shows that the cell shape was essentially unaffected by Camk2d deletion. DAPI staining was used for cell counting. ZO-1 intensity per cell and the cross sectional areas per cell were not different (Fig. 4*B*). The volume and surface area of nuclei in *Camk2d* KO clones were not significantly changed compared to the control clones (Fig. 4, *C* and *D*) although we cannot rule out a small effect. Phalloidin staining was carried out to assess the state of actin polymerization (Fig. 4*E*). No differences were seen between *Camk2d* KO and Ctrl clones. More specifically, dDAVP-induced actin depolymerization was similar in KO and Ctrl clones (Fig. 4, *E* and *F*).

### Proteomics and phosphoproteomics

**Figure 5:**
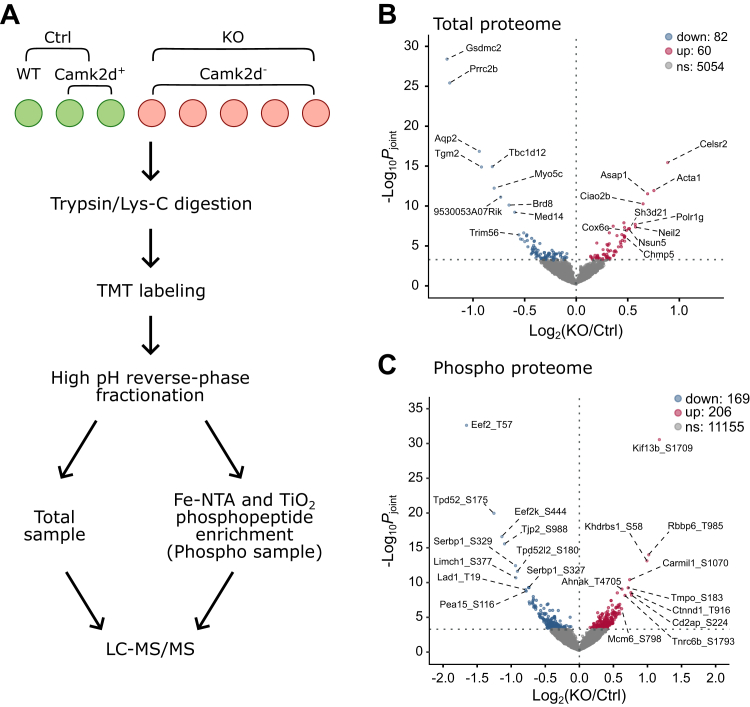
**Total and phospho-proteomics analysis of *Camk2d* KO versus *Camk2d* intact cells.***A*, the strategy for the sample preparation of total and phospho-proteomics by mass spectrometry. Cells were treated with dDAVP for short-term dDAVP (30 min, 0.1 nM). *B*, volcano plot of total proteome. One hundred forty-two proteins (60 with Log_2_(KO/Ctrl) >0 and 82 with Log_2_(KO/Ctrl) <0) underwent significant changes in response to dDAVP stimulation (*p*_joint_ < 0.0005). *C*, volcano plot of phospho-proteome. Three hundred seventy-five phosphosites (206 with Log_2_(KO/Ctrl) >0 and 169 with Log_2_(KO/Ctrl) <0) underwent significant changes in response to dDAVP stimulation (*p*_joint_ < 0.0005, see Experimental procedures). From the 11,570 identified phosphosites, phosphosites which underwent significant changes in total proteomics data were not included for *p*_joint_ calculation. CAMK2D, CAMK2δ; dDAVP, 1-desamino-8-D-arginine-vasopressin.

The main goal of this work is to identify phosphorylation sites whose phospho-occupancies are dependent on CAMK2D in mpkCCDc11-38 cells (called ‘mpkCCD cells’ from this point on). To do this, we carried out large-scale quantitative phosphoproteomic analysis using TMT labeling (Fig. 5*A*). In the TMT method, proteins from *Camk2d*-KO and *Camk2d*-intact (Ctrl) cells are separately labeled with unique mass-tags after trypsinization and combined into a single sample (see Experimental procedures). Thus, in each LC-MS/MS run, reporters for each of the original samples are quantified in the MS2 spectrum for each identified phospho-peptide. All cells were grown in the presence of dDAVP (0.1 nM present continuously in the growth medium until the day of the experiment). dDAVP was removed for 2 h and returned to the medium for 30 min. The relative amounts of a given phosphopeptide from *Camk2d*-KO and *Camk2d*-intact cells were calculated from their respective reporter intensities.

Figure 5*C* shows the phosphoproteomics results as a volcano plot. Approximately 3% of 11,570 phosphosites quantified underwent significant changes in the *Camk2d* KO cells, based on a relatively stringent criterion (*p*_joint_ < 0.0005, see Experimental procedures for *p*_joint_ definition). In total, 169 phosphosites were significantly decreased in abundance, while 206 phosphosites were increased. These data have been made available to users on a publicly accessible webpage allowing searching, browsing, and downloading of the results (https://esbl.nhlbi.nih.gov/Databases/CAMK2D-proteome/).

Total proteomics analysis was run on the same samples as for the phosphoproteomics but taken before the phosphopeptide enrichment step (Fig. 5*A*). The data are summarized as a so-called ‘volcano plot’ (Fig. 5*B*). The full data set is provided as Supporting Information. Only 0.3% of 5198 proteins underwent changes in total protein abundance. One of these is AQP2 which was less abundant in the KO samples (Total protein Log_2_(*Camk2d*-KO/*Camk2d*-intact) = −0.94).

**Figure 6:**
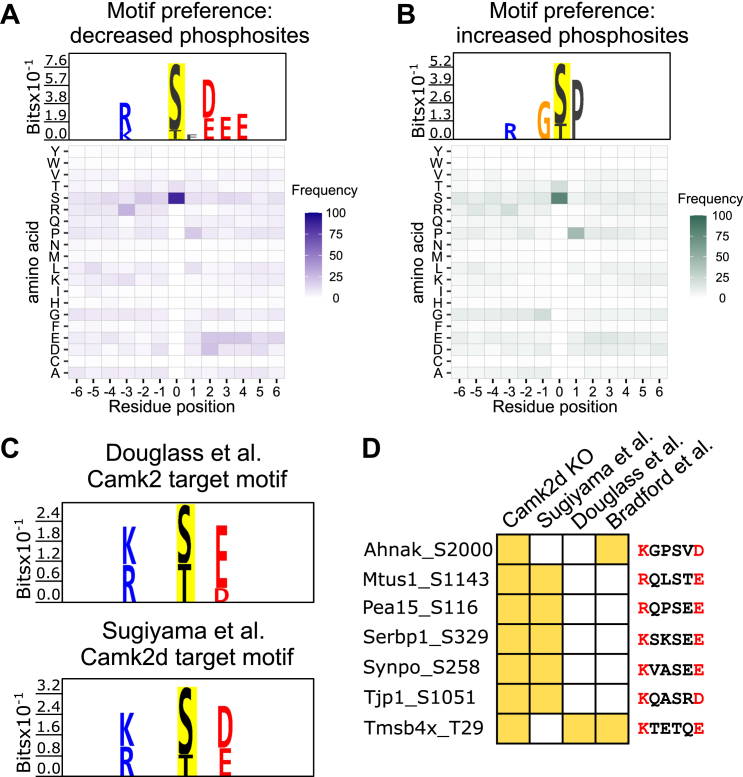
**Motif analysis based on the increased and decreased phosphosites of *Camk2d* KO cells.***A*, motif preference for the decreased phosphosites in *Camk2d* KO cells. Putative CAMK2D target site has R/K at position −3 and D/E at position +2. *B*, motif preference for the increased phosphosites in *Camk2d* KO cells shows preference for P at position +1. *C*, motif analysis from two previous *in vitro* phosphorylation studies (see text for references). In both studies, motif analysis showed that CAMK2 prefers R/K at position −3 and D/E at position +2. *D*, seven significantly decreased phosphosites with K/R at position −3 and D/E at position +2 were also identified in previous studies (see text for references). CAMK2, Ca2+/Calmodulin-dependent protein kinase 2; CAMK2D, CAMK2δ.

Figure 6*A* shows motif preference analysis for the downregulated phosphorylation sites. This analysis shows a preference for basic amino acids (lysine [K] and arginine [R]) in position −3 relative to the phosphorylated amino acid and a preference for acidic amino acids (aspartic acid [D] and glutamic acid [E]) in positions +2, 3, and 4. Target specificity for CAMK2D has been previously estimated using mass spectrometry in *in vitro* phosphorylation studies using recombinant CAMK2D to phosphorylate mixtures of proteins by Douglass *et al.* (31) and Sugiyama *et al.* (32). We calculated motifs from these two studies using *PTMLogo* (33). The CAMK2D motifs identified in these two studies are quite similar (Fig. 6*C*), closely matching the motif estimated in the present study in Figure 6*A* except that a preference for an acidic amino acid was only identified in position +2 and not +3 or +4. The inferred motif for CAMK2D is therefore (R/K)-X-X-p(S/T)-X-(D/E). A recent study using synthetic peptide arrays to identify kinase target motifs reported a very similar motif for the CAMK2 family (34). Focusing on the phosphorylation sites increased in response to *Camk2d* deletion, an entirely different motif was found (Fig. 6*B*). Because we deleted a protein kinase, the sites that were increased are necessarily indirect, for example, resulting from cascades of two or more kinases. The proline in position +1 is consistent with the activation of CMGC family kinases such as MAP kinases or cyclin-dependent kinases (34) or inhibition of a protein phosphatase. Among the CAMK2D target phosphosites, seven phosphosites were identified in the previous studies (ref), showing the analyzed motif sequence (Fig. 6*D*).

### Direct CAMK2D targets

By selecting the downregulated phosphorylation sites (*p*_joint_ < 0.0005 and Log_2_[*Camk2d*-KO/*Camk2d*-intact] < −0.3) after *Camk2d* deletion that also had the inferred CAMK2D motif (R/K)-X-X-p(S/T)-X-(D/E), we identified a set of 35 phosphorylation sites that are likely direct CAMK2D targets (Table 1).

**Table 1:** Direct phosphorylation targets for CAMK2D inferred from CRISPR-phosphoproteomics experiments

UniProt ID	Gene symbol	Annotation	Site	Centralized sequence^a^	Log_2_ (KO/Intact)	*p*_joint_
E9Q616	Ahnak	AHNAK nucleoprotein (desmoyokin)	S2000	GEL**K**GP**S**V**D**VEVP	−0.39	0.0001
A1IGU4	Arhgef37	Rho guanine nucleotide exchange factor 37	S15	ASS**K**SE**S**P**E**QEDQ	−0.38	0.0003
O88379	Baz1a	Bromodomain adjacent to zinc finger domain protein 1A	S1412	RGR**K**RQ**S**T**E**SSPV	−0.42	0.0001
Q9D219	Bcl9	B-cell CLL/lymphoma 9 protein	S104	KRE**R**SI**S**A**D**SFDQ	−0.33	0.0001
Q6EDY6	Carmil1	Leucine-rich repeat-containing protein 16A	S1328	SSP**R**SF**S**Q**E**ASRR	−0.31	0.0001
Q9JLQ0	Cd2ap	CD2-associated protein	S233	LRT**R**TS**S**S**E**TEEK	−0.52	0.0000
Q04899	Cdk18	Cyclin-dependent kinase 18	S66	QNQ**R**RF**S**M**E**DLNK	−0.33	0.0005
Q09XV5	Chd8	Chromodomain-helicase-DNA-binding protein 8	S2040	ARS**R**LT**S**Q**D**YEVR	−0.41	0.0005
Q8CJ61	Cmtm4	CKLF-like MARVEL transmembrane domain-containing protein 4	S194	IRA**R**TE**S**R**D**VDSR	−0.53	0.000
Q80XI3	Eif4g3	Eukaryotic translation initiation factor 4 gamma 3	S1151	TFL**R**GS**S**K**D**LLDN	−0.69	0.0000
Q05D44	Eif5b	Eukaryotic translation initiation factor 5B	S108	QKG**K**KT**S**F**D**ENDS	−0.43	0.0000
P18608	Hmgn1	Nonhistone chromosomal protein HMG-14	S7	MPK**R**KV**S**A**D**GAAK	−0.52	0.0000
Q6ZPV2	Ino80	DNA helicase INO80	S470	HQA**R**TR**S**F**D**EDAK	−0.43	0.0000
P15066	Jund	Transcription factor AP-1; transcription factor jun-D	S100	GLL**K**LA**S**P**E**LERL	−0.68	0.0000
Q5SVQ0	Kat7	Histone acetyltransferase KAT7	S228	CKV**R**AQ**S**R**D**KQIE	−0.46	0.0001
B1AVY7	Kif16b	Kinesin-like protein KIF16B	S838	QLV**K**LA**S**L**E**KDLV	−0.47	0.0003
P57016	Lad1	Ladinin-1	T19	SLA**R**QR**T**L**E**DEEE	−0.79	0.0000
Q3UH68	Limch1	LIM and calponin homology domains-containing protein 1	S379	SRR**R**SA**S**Q**D**LIKK	−0.58	0.0000
Q9Z2D1	Mtmr2	Myotubularin-related protein 2	S6	_ME**K**SS**S**C**E**SLGA	−0.33	0.0004
Q5HZI1	Mtus1	Microtubule-associated tumor suppressor 1 homolog	S1143	AIS**R**QL**S**T**E**QAAL	−0.45	0.0001
Q78HU3	Mvb12a	Multivesicular body subunit 12A	S168	QDM**R**GL**S**L**D**PPKE	−0.74	0.0000
Q9CX66	Nopchap1	NOP protein chaperone 1	S125	EMS**R**SD**S**K**E**EDSP	−0.46	0.0002
Q9DBS9	Osbpl3	Oxysterol-binding protein-related protein 3	S33	QGS**R**QD**S**W**E**VVEG	−0.40	0.0000
Q62048	Pea15	Astrocytic phosphoprotein PEA-15	S116	DII**R**QP**S**E**E**EIIK	−0.78	0.0000
Q9DBC7	Prkar1a	cAMP-dependent protein kinase type I-alpha regulatory subunit	S77	TGI**R**TD**S**R**E**DEIS	−0.36	0.0003
Q6GYP7	Ralgapa1	Ral GTPase-activating protein subunit alpha-1	T753	SIV**R**QK**T**V**D**IDDA	−0.39	0.0002
Q8BU14	Sec62	Translocation protein SEC62	S309	KQQ**K**SD**S**E**E**KSDS	−0.40	0.0003
Q9CY58	Serbp1	Plasminogen activator inhibitor 1 RNA-binding protein	S329	VLH**K**SK**S**E**E**AHAE	−0.94	0.0000
Q6ZWQ0	Syne2	Nesprin-2	T6489	LLL**R**QG**T**D**D**SKEG	−0.65	0.0000
Q8CC35	Synpo	Synaptopodin	S258	HLE**K**VA**S**E**E**EEVP	−0.54	0.0000
P39447	Tjp1	Tight junction protein ZO-1	S1051	YIE**K**QA**S**R**D**LEQP	−0.63	0.0000
Q9Z0U1	Tjp2	Tight junction protein ZO-2	S988	SQN**R**ED**S**F**D**YSKS	−1.10	0.0000
P20065	Tmsb4x	Thymosin beta-4; Hematopoietic system regulatory peptide	T29	KLK**K**TE**T**Q**E**KNPL	−0.64	0.0000
Q99PP7	Trim33	E3 ubiquitin-protein ligase TRIM33	S1134	RRK**R**LK**S**D**E**RPVH	−0.37	0.0003
Q8BJ05	Zc3h14	Zinc finger CCCH domain-containing protein 14	S309	VKV**K**RF**S**H**D**GEEE	−0.38	0.0004

*p*_joint_ is the product of the *p* value from T-statistic and the Gaussian probability (see Experimental procedures).

a Central S or T is the identified phosphorylation site. All sequences match -(R/K)-X-X-p(S/T)-X-(D/E) motif determined from *in vitro* phosphorylation studies.

**Figure 7:**
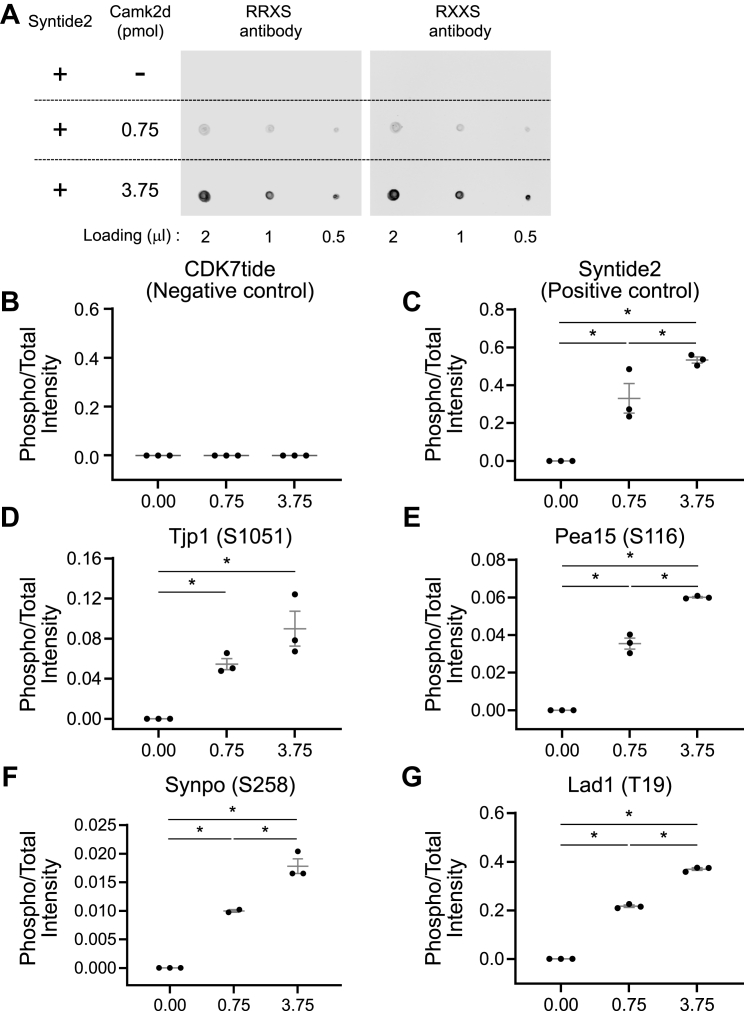
***In vitro* phosphorylation.***A*, dot blot of Syntide2. Syntide2 which is a known Camk2d target peptide was phosphorylated in the presence of CAMK2D. *B*–*G*, the ratio of phospho to total intensity obtained from the proteomics data. CDK7tide (negative control) was not phosphorylated, and syntide2 (positive control) was significantly phosphorylated by CAMK2D. Tjp1 (S1051), Pea15 (S116), Synpo (S258), and Lad (T19) were significantly phosphorylated in the presence of CAMK2D while those peptides were not phosphorylated in the absence of CAMK2D. ∗ indicates significance (*p* < 0.05), one-way ANOVA test. Samples were triplicated. Intensity values calculated in MaxQuant. CAMK2D, CAMK2δ.

To confirm that CAMK2D can indeed phosphorylate novel sites identified using the CRISPR-phosphoproteomics methodology, we performed *in vitro* phosphorylation experiments using quantitative protein mass spectrometry to measure phosphorylation of selected target sites alter incubation with varying concentrations of recombinant CAMK2D. Out of the 35 peptides listed, we selected six peptides [Jund (S100), Lad1 (T19), Mvb12a (S168), Pea15 (S116), Synpo (S258), and Tjp1 (S1051)] for these validation experiments. To confirm the activity of the recombinant CAMK2D, dot blotting was performed (Fig. 7*A*), which showed that syntide2 (positive control) was phosphorylated in the presence of 0.75 or 3.75 pmol of CAMK2D; in contrast, in the absence of CAMK2D, syntide2 remained unphosphorylated. With the quantitative mass spectrometry readout, syntide2 exhibited substantial phosphorylation in a CAMK2D dose-dependent manner, whereas CDK7tide (negative control peptide) did not undergo phosphorylation by CAMK2D (Fig. 7, *B* and *C*). Among the six selected peptides, four peptides were significantly phosphorylated in the presence of CAMK2D but were not phosphorylated in the absence of CAMK2D (Fig. 7, *D*–*G*).

The 35 newly identified targets compares with 21 phosphoproteins previously identified as CAMK2D targets from reductionist studies as reported at PhosphoSitePlus (https://www.phosphosite.org/). These phosphoproteins were ANKRD28 (35), BMAL1 (36, 37), CACNB1 (38, 39), CACNB2 (39, 40), CAMK2D (41, 42, 43), CEACAM1 (44), GLO1 (45, 46), HDAC4 (47, 48), HDAC5 (47, 49), ITPR2 (50), KCNJ11 (51), KCNQ1 (52), MYBPC3 (53), OCLN (54), PLN (55), PPP3CA (56), RYR2 (57, 58), SCN5A (59), STX3 (60), TTN (61), TPD52 (62). Other CAMK2 family members have been noted to phosphorylate other targets (3). Thus, the present study has more than doubled the number of known targets of CAMK2D. We mapped the 35 new sites to cellular functions regulated in collecting duct cells in response to vasopressin in Table 2. The largest groups map to “altered membrane trafficking of AQP2” and “increased *Aqp2* gene transcription”, providing new hypotheses for proteins involved in vasopressin-mediated regulation of these processes.

**Table 2:** Mapping of Camk2d target phosphoproteins to vasopressin-regulated cellular processes

Vasopressin-regulated cellular process	References	Phosphoproteins targeted by CAMK2D
Altered membrane trafficking of AQP2	(73, 74, 75, 76)	Arhgef37, Cd2ap, Cdk18, Cmtm4, Kif16b, Mtmr2, Mvb12a
Increased *Aqp2* gene transcription	(30, 77)	Baz1a, Bcl9, Chd8, Hmgn1, Ino80, Jund, Kat7, Ralgapa, Tmsb4x
Increased DNA accessibility at *Aqp2* gene	(87)	Baz1a, Chd8, Hmgn1, Ino80, Kat7, Trim33
Increased AQP2 translation	(78)	Eif4g3, Eif5b, Sec62
Increased AQP2 protein half life	(78, 88)	Mvb12a, Trim33
Intracellular calcium mobilization	(10, 24, 25)	none
Reorganization of actin filaments	(9, 20, 21, 23)	Cd2ap, Lad1, Limch1, Syne2, Synpo, Tmsb4x
Depolymerization of filamentous actin	(20, 21, 22)	Carmil1
Microtubule-dependent AQP2 redistribution	(89)	Ino80, Kif16b, Mtus1, Syne2
Increased tight junction permeability	(90)	Ahnak, Cd2ap, Synpo, Tjp1, Tjp2
Decreased rate of apoptosis	(91)	Chd8, Serbp1
Decreased rate of proliferation	(92)	Hmgn1, Jund, Kat7, Pkar1a
Increased principal cell size	(11, 93, 94, 95)	none
Decreased ERK1/2 activity	(18, 96)	Pea15

### Downstream kinases

Table 3 shows all protein kinases that underwent changes in phosphorylation in response to deletion of *Camk2d*. Two of these are proline-directed kinases, namely CDK18 and CDK1. The CDK18 site (Ser66) displayed the (R/K)-X-X-p(S/T)-X-(D/E) characteristic of CAMK2D. The phosphorylation decreases significantly, but the effect on kinase activity is unknown. If the change in phosphorylation results in an increase in kinase activity, it could be responsible for the overall increase in phosphorylation of sites with P in position +1 (Fig. 5*B*). Cdk1 undergoes an increase in phosphorylation at two neighboring sites that are well characterized (T14 and Y15) (63, 64, 65). Phosphorylation at these sites inhibits kinase activity (63, 65, 66) and so this kinase is unlikely to play a role in the overall increase in phosphorylation of sites with P in position +1. Thus, the pathway resulting in activation of these sites remains uncertain.

**Table 3:** Downstream protein kinases that underwent changes in phosphorylation by CAMK2D deletion

UniProt ID	Gene symbol	Annotation	Site	Centralized sequence	Log_2_ (KO/Intact)	*p*_joint_	Domain
Q9Z277	Baz1b	Tyrosine-protein kinase BAZ1B	S325	KKPKRDSSSLSSP	−0.124	0.0002	none
P11440	Cdk1	Cyclin-dependent kinase 1	T14	EKIGEGTYGVVYK	0.525	0.0000	catalytic
P11440	Cdk1	Cyclin-dependent kinase 1	Y15	KIGEGTYGVVYKG	0.461	0.0000	catalytic
Q04899	Cdk18	Cyclin-dependent kinase 18	S66	QNQRRFSMEDLNK	−0.332	0.0005	none
O08796	Eef2k	Eukaryotic elongation factor 2 kinase	S444	GDSGYPSEKRSDL	−1.136	0.0000	none
P70424	Erbb2	Receptor tyrosine-protein kinase erbB-2	Y878	DIDETEYHADGGK	−0.190	0.0001	catalytic
Q9ESL4	Map3k20	Mitogen-activated protein kinase kinase kinase MLT	S567	PGSRSDSSADCQW	0.371	0.0002	none
Q9ESL4	Map3k20	Mitogen-activated protein kinase kinase kinase MLT	S638	NPSRSSSPTQYGL	0.432	0.0000	none
Q8BPM2	Map4k5	Mitogen-activated protein kinase kinase kinase kinase 5	S433	QVLRRQSSPSCVP	−0.450	0.0000	none
Q3U214	Mast3	Microtubule-associated serine/threonine-protein kinase 3	S702	RYRHLGSEDDETN	0.434	0.0001	AGC-kinase C-terminal
Q6P9R2	Oxsr1	Serine/threonine-protein kinase OSR1	S359	AISQLRSPRVKDS	0.450	0.0000	none
Q8CIN4	Pak2	Serine/threonine-protein kinase PAK 2	T143	QKYLSFTPPEKDG	−0.462	0.0004	none
Q8CEE6	Pask	PAS domain-containing serine/threonine-protein kinase	S852	ILHRQTSDILVDR	0.519	0.0000	none
Q5EG47	Prkaa1	5′-AMP–activated protein kinase catalytic subunit alpha-1	S508	SCQRSDSDAEAQG	−0.306	0.0004	none
Q5EG47	Prkaa1	5′-AMP–activated protein kinase catalytic subunit alpha-1	T490	AKSGTATPQRSGS	0.366	0.0000	none
P97313	Prkdc	DNA-dependent protein kinase catalytic subunit	S3215	VDEDEESIDREVY	0.291	0.0001	FAT
O54988	Slk	STE20-like serine/threonine-protein kinase	S779	KDSGSVSLQETRR	0.511	0.0000	none
O55098	Stk10	Serine/threonine-protein kinase 10	S13	RRILRLSTFEKRK	0.469	0.0001	none
Q9WTK7	Stk11	Serine/threonine-protein kinase	S31	FIHRIDSTEVIYQ	−0.275	0.0004	none

### Downstream phosphatases

Secondary effects of *Camk2d* deletion could also be due to downstream regulation of protein phosphatase activities. Table 4 shows protein phosphatase subunits that underwent changes in phosphorylation in *Camk2d* deletion clones versus controls. There were two sites in the regulatory subunit of the protein phosphatase PPP1C (PPP1R12A) that underwent increases in phosphorylation. One of these sites, Ser445, has been shown to inhibit this subunit from binding to phosphatases through 14-3-3 interactions (67). Thus the demonstrated increase in PPP1R12A phosphorylation at Ser445 would be predicted to inhibit phosphatase activity, possibly explaining increases in phosphorylation at some sites. Another phosphatase regulator, PPP1R2 functions as a phosphatase inhibitor. It undergoes a decrease in phosphorylation at Ser90, with unknown effect on activity. An additional phosphatase, SSH1 or Slingshot 1, is a protein tyrosine phosphatase with uncertain role in CAMK2D signaling.

**Table 4:** Downstream protein phosphatases and phosphatase-related proteins that underwent changes in phosphorylation by CAMK2D deletion

UniProt ID	Gene symbol	Annotation	Site	Centralized sequence	Log_2_ (KO/Intact)	*p*_joint_
Q9DBR7	Ppp1r12a	Protein phosphatase 1 regulatory subunit 12A	S445	GLRKTGSYGALAE	0.305	0.0000
Q9DBR7	Ppp1r12a	Protein phosphatase 1 regulatory subunit 12A	T669	RRRSYLTPVRDEE	0.318	0.0002
Q9DCL8	Ppp1r2	Protein phosphatase inhibitor 2	S90	EDAYSDSEGNEVM	−0.446	0.0001
Q76I79	Ssh1	Protein phosphatase Slingshot homolog 1	T981	GSLNFSTEDLSSE	0.305	0.0004

### Bioinformatics

We also carried out an unbiased bioinformatic analysis using DAVID 2021 to identify *Gene Ontology Biological Process (GO-BP)* terms that are associated with altered phosphoproteins. The phosphoproteins associated with downregulated phosphorylation sites are shown in Table 5. The direct CAMK2D targets are shown in bold. The major enriched GO-BP terms include several associated with processes relevant to AQP2 regulation including regulation of actin cytoskeleton, epithelial polarity determination and differentiation, apoptosis, TGF-beta signaling, regulation of transport, and endocytosis. The phosphoproteins associated with upregulated phosphorylation sites are shown in Table 6. This includes many of the same processes seen for the downregulated sites but also includes GO terms regulated to DNA replication gene expression and RNA processing. The major enriched GO-BP terms were those associated with processes relevant to AQP2 regulation including those pertaining to regulation of gene expression, DNA replication, cell polarity, RNA splicing, microtubule organization, and endocytosis.

**Table 5:** List of *Gene Ontology Biological Process* terms enriched among phosphoproteins with phosphorylation sites decreased by CAMK2D deletion∗

Term (count)	Fold enrichment	*p* (Fisher exact)	Gene symbol
Establishment of monopolar cell polarity (4)	12.50	0.000	Scrib, Camsap3, Slc9a3r1, Map1b
Actin cytoskeleton organization (17)	2.37	0.001	**Prkar1a**, Lima1, Arhgef2, Kank3, Vasp, Snx9, **Synpo**, Fhod1, Slc9a3r1, **Tjp1**, **Carmil1**, Shroom2, **Cd2ap**, **Limch1**, Srcin1, Pak2, Marcksl1
Establishment or maintenance of apical/basal cell polarity (4)	6.48	0.003	Pard3, Scrib, Camsap3, Slc9a3r1
Regulation of apoptotic process (23)	1.78	0.004	Niban2, Tpd52l1, Scrib, **Chd8**, Wfs1, Gapdh, Slc9a3r1, Prkaa1, Mff, Erbb2, Casp3, **Tjp1**, Akap12, Zfand6, **Pea15**, Eef2k, Oxr1, Acin1, Suds3, **Serbp1**, Pla2g4a, Rybp, Pak2
Regulation of transforming growth factor beta receptor signaling pathway (5)	4.29	0.006	**Trim33**, Bcl9l, Itga3, Suds3, Veph1
Negative regulation of microtubule depolymerization (3)	6.91	0.009	Arhgef2, Camsap3, Map1b
Positive regulation of transport (14)	1.80	0.022	**Prkar1a**, Asph, Wfs1, **Mtmr2**, Slc9a3r1, Ubr5, Prkaa1, Mff, Erbb2, Emd, Eef2k, Pard3, **Cd2ap**, Pla2g4a
Epithelial cell differentiation (10)	1.98	0.029	Pard3, Scrib, Tjp3, Camsap3, Fndc3a, Slc9a3r1, **Tjp1**, Casp3, **Tjp2**, Map1b
Endocytosis (11)	1.87	0.032	**Carmil1**, Eef2k, Pard3, Scrib, Snx9, Fkbp15, **Cd2ap**, Lyst, **Mtmr2**, Mff, Dennd1b

Bold indicates inferred direct phosphorylation site.

∗ Based on analysis using DAVID 2021.

**Table 6:** List of *Gene Ontology Biological Process* terms enriched among phosphoproteins with phosphorylation sites increased by CAMK2D deletion∗

Term (count)	Fold enrichment	*p* (Fisher exact)	Gene symbol
DNA replication (11)	3.11	0.001	Mcm6, Nfib, Ssrp1, Mcm3, Lig1, Ttf1, Purb, Rbbp6, Cdk1, Supt16 h, Cdt1
Establishment of monopolar cell polarity (3)	8.01	0.006	Patj, Camsap3, Gbf1
Cell development (31)	1.57	0.006	Micall1, Camsap1, Crebbp, Nfib, Zbtb7a, Pard3, Septin7, Atn1, Kmt2d, Kif13b, Snx1, Shc1, Pdlim5, Pdcd4, Mia3, Camsap3, Prkdc, Nolc1, Nfatc2, App, Mrtfb, Slc44a4, **Carmil1**, Rbm10, Tcof1, Brd1, Wasl, Adgrg6, Nrdc, Myo1e, Ccm2
Regulation of RNA splicing (8)	2.74	0.008	Rbm10, Srsf7, Q3TQI7, Nup98, **Ahnak**, Khdrbs1, Fxr2, Zbtb7a
Cytoplasmic microtubule organization (4)	4.27	0.013	Camsap1, Camsap3, Clasp1, Slk
Actin cytoskeleton organization (15)	1.79	0.020	Pdlim5, Sptbn1, Epb41l1, Arhgap17, Clasp1, Mrtfb, Cald1, **Carmil1**, Washc2, Sptbn2, Wasl, **Cd2ap**, Shc1, Myo1e, Epb41l2
Golgi vesicle transport (10)	2.03	0.025	Nbas, Eps15, Plcb3, Snx2, Mia3, Mia2, Sptbn1, Snx1, Arcn1, Gbf1
Regulation of gene expression (48)	1.27	0.030	Znf740, Srsf7, Zc3h18, Crebbp, Tcf20, Cxxc1, Nfib, Cnpy2, Purb, Pelp1, Tmpo, Fxr2, Cdk1, Eif4ebp2, Zbtb7a, Supt16 h, Akap8l, Atn1, Kmt2d, Cdc5l, Khdrbs1, Rsf1, Rai1, Shc1, Phip, Ctr9, Nab2, Q3TQI7, Hnrnpa1, Pdcd4, Ctnnd1, Nup98, Prkdc, Cic, Nolc1, Nfatc2, Prkaa1, App, Mrtfb, Eif3b, Nbas, Pkp3, Rbm10, Tcof1, Zzz3, Tnrc6b, Wasl, **Ahnak**
RNA processing (23)	1.48	0.033	Ctr9, Prpf38b, Srsf7, Sf3a3, Q3TQI7, Srrm2, Hnrnpa1, Nup98, Prkdc, Pnn, Dbr1, Pelp1, Rbbp6, Fxr2, App, Zbtb7a, Akap8l, Rbm10, Ints1, Cdc5l, Khdrbs1, Bysl, Trmt10a
Endocytosis (12)	1.74	0.041	Eps15, **Carmil1**, Pard3, Micall1, Snx2, Wasl, **Cd2ap**, Cnpy2, Snx1, Myo1e, Fcho2, App
Mitotic cell cycle process (15)	1.62	0.043	Map3k20, Smc3, Mcm6, Crebbp, Prkdc, Sptbn1, Clasp1, Lig1, App, Cdk1, Cdt1, Akap8l, Septin7, Mcm3, Phip

Bold indicates inferred direct phosphorylation sites.

∗ Based on analysis using DAVID 2021.

### RNA sequencing

**Figure 8:**
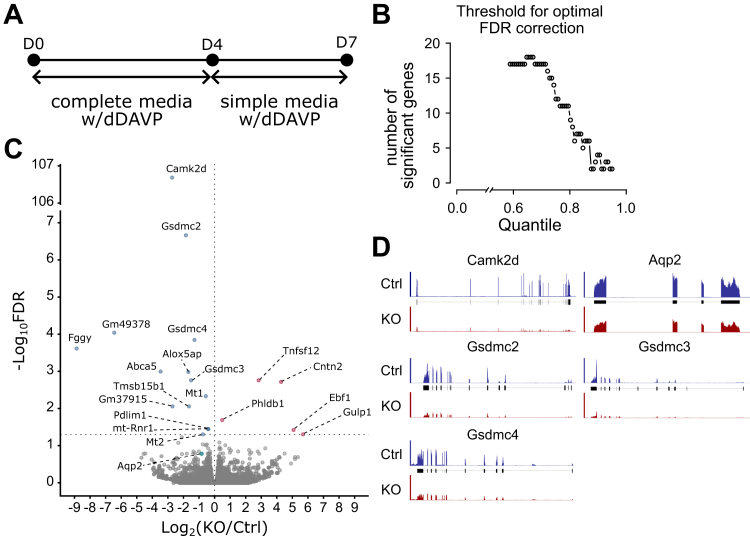
**RNA-seq–based transcriptomic analysis of *Camk2d* KO versus *Camk2d* intact cells.***A*, cell culture strategy for long-term dDAVP (7 days, 0.1 nM) stimulation. *B*, plot for the cut-off threshold to determine the optimal false discovery rate (FDR) correction. The cut-off threshold was calculated, and it was determined by the value which assigned the most significant genes. *C*, volcano plot of RNA-seq. Only 19 transcripts underwent significant changes (14 decreased genes and 5 increased genes). *D*, distribution of RNA-seq reads across gene bodies of selected genes: *Camk2d* (decreased), *Gsdmc2* (decreased), *Gsdmc3* (decreased), *Gsdmc4* (decreased), *AQP2* (unchanged). AQP2, aquaporin-2; CAMK2D, CAMK2δ; dDAVP, 1-desamino-8-D-arginine-vasopressin.

We also ran a transcriptomic analysis on *Camk2d*-KO versus *Camk2d*-intact mpkCCD cell lines using RNA-seq (Fig. 8). This analysis showed that only a very small fraction of total quantified transcripts underwent significant changes in expression (19 of approximately 20,000 transcripts which include products of both protein-coding and noncoding genes). Because so few transcripts were changed, gene enrichment analysis was not possible. However, standing out among the 19 transcripts that underwent changes were three gasdermin genes (*Gsdmc2, Gsdmc3*, and *Gsdmc4*), pore-forming proteins that cause membrane permeabilization and pyroptosis (68). Gasdermins have also been seen to play additional roles in cells involved in the regulation of apoptosis, signaling, mitochondrial function, and protein translation (68).

## Discussion

In this study, we combined CRISPR-Cas9–mediated genome editing with phosphoproteomic analysis to identify direct and indirect targets of CAMK2D in renal collecting duct cells. The analysis, when coupled with prior *in vitro* phosphorylation data, identified 35 novel phosphorylation sites that appear to be direct CAMK2D targets. Additional phosphorylation sites that underwent changes in abundance are likely to be indirect targets, which are phosphorylated by other kinases that are in turn regulated by CAMK2D. The method employing CRISPR-Cas9 coupled to phospho-proteomics for identification of novel protein kinase targets has previously been used successfully to identify target sites for PKA (18) and MLCK (28). These studies markedly expanded the lists of known targets for these protein kinases. The findings in this study are relevant both to the regulation of the water channel AQP2 in the collecting duct and potentially to CAMK2D targets in other tissues. The full CRISPR-phosphoproteomic dataset is provided to readers as a publicly accessible web resource allowing searching, browsing, and download of data for further interpretation (https://esbl.nhlbi.nih.gov/Databases/CAMK2D-proteome/). Other methods have been employed to carry out large-scale identification of protein kinase targets, most notably the use of kinase selective inhibitors coupled with phosphoproteomics (69, 70).

The 35 direct targets all possessed the CAMK2 consensus sequence (R/K)-X-X-p(S/T)-X-(D/E) and showed statistically significant decreases in phospho-occupancy in multiple *Camk2d*-KO cell lines versus *Camk2d*-intact cells. All are novel targets, adding to the list of 21 target phosphoproteins previously identified in reductionist studies and cataloged at PhosphoSitePlus (https://www.phosphosite.org/). Four of the 35 newly identified phosphorylation sites were confirmed as CAMK2D targets in *in vitro* phosphorylation studies using recombinant CAMK2D, further validating the CRISPR-phosphoproteomics methodology. Most of the 21 previously identified phosphoproteins are widely expressed among tissues and are likely targets outside of the kidney. The consensus sequence (R/K)-X-X-p(S/T)-X-(D/E) was determined from data reported in prior studies in which recombinant protein kinases were used either for *in vitro* phosphorylation using arrays of synthetic target peptides (34) or mixtures of dephosphorylated proteins followed by protein mass spectrometry (31, 32). In addition, many of the known phosphorylation targets of CAMK2D from reductionist studies reported at *PhosphoSitePlus* have this motif.

The current experiments also identified phosphorylation events that are indirectly mediated by CAMK2D, which would not be detected in an *in vitro* system. Many of these phosphoproteins had sites that were increased in phospho-occupancy, which is necessarily an indirect effect of the *Camk2d* deletion. These upregulated sites had a preponderance of sequences with proline (P) in position +1 consistent with CMGC family kinases that include both MAP kinases and cyclin-dependent kinases (34). Among this group of kinases, only one was found to be a direct target of CAMK2D, namely CDK18 at Ser66 (centralized sequence: QNQRRFpSMEDLNK). This site has been previously labeled as a PKA target site, but the effect of phosphorylation on activity is unclear (71). Another possibility for the increase in phosphorylation at sites with proline in position +1 is that a proline-directed phosphatase like PP2A (72) may be inhibited downstream from CAMK2D. This study does not provide evidence for such a possibility. There were several protein kinases that underwent changes in phosphorylation in response to *Camk2d* deletion and these likely mediate some of the indirect effects of *Camk2d* deletion.

What do these results tell us about vasopressin action and AQP2 regulation? Vasopressin increases calcium mobilization in collecting duct principal cells (10, 24, 25) which activates calmodulin-dependent kinases including CAMK2D. AQP2 is regulated in multiple ways, namely regulated trafficking to and from the apical plasma membrane of collecting duct cells (73, 74, 75, 76) and regulation of AQP2 protein abundance by transcriptional (30, 77) and posttranscriptional (78) mechanisms (Table 2). Calmodulin inhibitors rapidly reduced osmotic water permeability in isolated perfused collecting ducts (10) presumably through effects on AQP2 trafficking, which is regulated in part through AQP2 phosphorylation in a cluster of four serines in the C-terminal tail (79). One of these, Ser256, was proposed to be phosphorylated by CAMK2D (4, 8). Note that Ser256 is within an amino acid sequence (VRRRQpSVELHS) that corresponds to the inferred CAMK2D target motif. Indeed, immunoblotting showed that deletion of CAMK2D decreased AQP2 phosphorylation at Ser256 even when the observed reduction in total AQP2 was taken into account. Another AQP2 phosphorylation site, Ser269, is believed to regulate AQP2 endocytosis (80, 81, 82). Immunoblotting also showed a decrease in Ser269 phosphorylation in *Camk2d* KO cells (Fig. 2). Consistent with the changes in Ser269 phosphorylation, the *Gene Ontology* analysis identified ‘Endocytosis’ as an enriched biological process. However, AQP2 internalization after dDAVP washout appeared to be unaffected by Camk2d deletion.

AQP2 protein abundance was decreased in the *Camk2d* KO cell lines, although the abundance of the corresponding AQP2 transcript was not significantly decreased. Very few transcript abundances changed and there was no overlap with the response to deletion of PKA with the exception of gasdermin C, a pyroptosis effector protein.

Vasopressin also regulates the actin cytoskeleton resulting in F-actin depolymerization in the apical region (20, 21, 22) (Table 2). Indeed the *Gene Ontology Biological Process* term ‘Actin Cytoskeleton Organization” showed an association with phosphoproteins that underwent changes in phosphorylation with *Camk2d* deletion. This includes phosphorylation changes in *Arhgef2, Cd2ap, Pak2*, *and Lima1* (EPLIN). Nevertheless, there was no obvious change in F-actin distribution in the cells as assessed by phalloidin staining. However, it is possible that there were localized effects not detectable by the method employed.

Another action of vasopressin in collecting duct cells is regulation of cell junctions (Table 2). Indeed, both TJP1 (ZO-1) and TJP2 (ZO-2) had significant decreases in phosphorylation at putative Camk2d sites in response to *Camk2d* deletion. However, *Camk2d* deletion did not alter transepithelial resistance (Fig. 2). In addition, there was no detectable change in ZO-1 distribution or abundance in *Camk2d* KO cells (Fig. 3). In addition, two of the enriched *Gene Ontology Biological Process* terms are related to cell polarity. For example, there were multiple distinct PDZ domain proteins that underwent phosphorylation changes, namely SLC9A3R1, SCRIB, and PARD3, as well as the aforementioned ZO-1 and ZO-2.

In general, although the phosphorylation changes seen with *Camk2d* deletion pointed to specific physiological processes, we did not see strong effects on these processes. This could be due in part to the redundancy in the CAMK family. Table 7 shows the expression of CAMK2 family transcripts and proteins in collecting duct. The transcriptomics in the present study shows significant expression of two isoforms other than *Camk2d*, namely *Camk2b* and *Camk2g*. However, in proteomic analysis, CAMK2A and CAMK2B were undetectable and the CAMK2G abundance was only about 7% of the CAMK2D value. Also, the data in Table 7 did not show evidence of compensation, that is an increase in the nondelta isoforms in *Camk2d* KO. Thus, it is conceivable that these additional family members are able to maintain the aforementioned physiological functions. Note also that even the inferred direct targets of CAMK2D sustained some residual phosphorylation in the *Camk2d* KO cells, supporting the hypothesis that there may be redundancy in CAMK2 function in collecting duct cells.

**Table 7:** Expression of Camk2 isoforms in response to Camk2d deletion

RNA seq					
Gene symbol	Annotation	Intact mean TPM	KO mean TPM	Log_2_ KO/Intact	FDR
Camk2a	Calcium/calmodulin-dependent protein kinase II alpha	0.023	0.031	0.767	0.998
Camk2b	Calcium/calmodulin-dependent protein kinase II, beta	3.517	3.646	0.120	0.998
Camk2d	Calcium/calmodulin-dependent protein kinase II, delta	7.059	4.671	−2.725	0.000
Camk2g	Calcium/calmodulin-dependent protein kinase II gamma	2.585	2.438	−0.029	0.998


Total protein changes					
Gene symbol	Annotation	Intact mean intensity	KO mean intensity	Log_2_ KO/Intact	*p*_joint_
Camk2a	Calcium/calmodulin-dependent protein kinase II alpha	N.D.	N.D.		
Camk2b	Calcium/calmodulin-dependent protein kinase II, beta	N.D.	N.D.		
Camk2d	Calcium/calmodulin-dependent protein kinase II, delta	1,003,719	163,702	−2.617	9.9E-120
Camk2g	Calcium/calmodulin-dependent protein kinase II gamma	74,141	58,360	−0.389	0.0001

N.D., not detectable.

## Experimental procedures

### Cell culture

Studies were done in a mice cortical collecting duct cell line (mpkCCDc11 secondary clone 38, mpkCCD_C11-38_) which was recloned from mpkCCDc11 cells (28). The cells were grown on permeable membrane supports (Cat. No. 3460, 3450 and 3419, Corning) in complete DMEM/Ham’s F12 medium (DMEM/F12) containing 5 μg/ml insulin, 50 nM dexamethasone, 1 nM triiodothyronine, 10 ng/ml epidermal growth factor, 60 nM sodium selenite, 5 μg/ml transferrin, and 2% fetal bovine serum in the presence of 0.1 nM dDAVP (V1005, Sigma) for 4 days. Then, the medium was changed to a simple serum-free/growth factor-free medium (with 50 nM dexamethasone, 60 nM sodium selenite, 5 μg/ml transferrin) with 0.1 nM dDAVP for 3 days to ensure complete polarization and cell differentiation. Complete medium was changed every other day, and simple medium was changed every day. For short-term experiments, dDAVP was removed for 2 h and then cells were exposed to either vehicle or 0.1 nM dDAVP on the basolateral side for 30 min. For long-term experiments, after 4 days growth in complete medium, cells were treated with vehicle or 0.1 nM dDAVP on the basolateral side for 3 days in simple medium.

### Generation of Camk2d-Null and Camk2d-Intact cell lines

mpkCCD_C11-38_ cells were transfected with CMV-eCas9-2a-tGFP plasmids (MMPD0000110416, MMPD0000110419, Sigma) containing either of two gRNAs specific for *Camk2d* gene, using Lipofectamine 3000 (L3000015, Invitrogen) according to the manufacturer’s instructions. The sequence of the two gRNAs are gRNA1; 5′-TCCATAAGGATCTTTACGC-3′, gRNA2; 5′- GATCTTCATCCCAGAAGGG-3’. The GFP-expressing cells were sorted into 96-well plates (∼1 cell per well) using a BD FACSAria Fusion Flow Cytometer. These cells were cultured for 2 to 3 weeks and amplified for the further investigation. The expression of CAMK2D was evaluated by semiquantitative immunoblotting. The genomic indel mutations were identified by Sanger sequencing. Also, *Camk2d*-intact (control) clones which were transfected with the plasmid but continued to express high levels of CAMK2D protein were without any mutation as evaluated by Sanger sequencing. Five separate KO clones were used as biological replicates in subsequent experiments. Two clones that underwent the transfection procedure but retained CAMK2D expression were chosen as Control (Ctrl) clones (considered biological replicates). We retained the one parental line as an additional control (giving a total of three control biological replicates).

### Genomic sequencing

Genomic DNA of *Camk2d* KO clones was extracted using DNeasy Blood & Tissue Kit (69504, Qiagen). Approximately 300 bp and 500 bp region flanking the gRNA target sites were amplified using PCR Master Mix (K0171, Thermo Fisher Scientific). The amplified PCR fragments were further inserted in pCR4-TOPO plasmid (450030, TOPO TA Cloning Kit, Invitrogen) and cloned using DH5α competent cells. At least five colonies were picked for Sanger sequencing. Primer sequences for two gRNAs were as follows: 1) forward primer: 5′- GCATGTGCACACACCCTTTC-3′, reverse primer: 5′- CACCACGGGTGAACTCAAAAC-3′, 2) forward primer: 5′- CCACAGGATGGCCTGACAAT-3′, reverse primer: 5′- CCAACCAACCCTCATCCCAA-3′.

### Immunoblotting, dot blotting, and antibodies

For immunoblotting, the cells were rapidly washed with ice-cold Dulbecco's phosphate-buffered saline, and lysis buffer (1.5% SDS, 10 mM Tris, pH 6.8) containing protease and phosphatase inhibitor (78441, Thermo Fisher Scientific) was added to solubilize the cellular proteins. The denatured protein samples were subjected to SDS-PAGE using 4 to 20% or 12% Criterion TGX Gels (5671095, 5671045, Bio-Rad). Proteins were transferred to nitrocellulose membranes, blocked, and probed with primary antibodies. For dot blotting, the reaction mixtures of *in vitro* phosphorylation were spotted 2, 1, and 0.5 μl onto the nitrocellulose membrane. The membrane was incubated at room temperature (RT) for 30 min to dry the membrane, followed by blocked and probed with primary antibodies. Primary antibodies were anti-AQP2 (K5007, 1:5000) (83), anti-phospho-S256 AQP2 (1:1000) (84), anti-phospho-S269 AQP2 (1:1000) (83), anti-Camk2d (ab181052, Abcam, 1:1000), anti-phospho-PKA substrate (RRXS/T) (9624S, CST, 1:1000), and anti-Phospho-(Ser/Thr) PKA substrate (RXXS/T) (9621S, CST, 1:1000). Blocking buffer and infrared fluorescence-conjugated secondary antibodies were obtained from Li-COR. Fluorescence images were visualized by the Li-COR Odyssey System (ODY-0428). Band intensities were analyzed using Li-COR Image Studio software (https://www.licor.com/bio/image-studio/).

### Immunofluorescence microscopy

Cells were prepared with the short-term dDAVP protocol as described above (Cell culture section). Briefly, after 30 min incubation with dDAVP, cells were fixed with 4% paraformaldehyde for 10 min at RT. Cells were permeabilized with permeabilizing solution (0.3% Triton X-100 and 0.1% bovine serum albumin in PBS) for 10 min and washed three times, followed by blocking with blocking solution (1% bovine serum albumin and 0.2% gelatin in PBS) for 30 min. Cells were incubated with primary antibodies overnight at 4 °C. Anti-ZO-1 (339194, Invitrogen) and phalloidin (A12380, Invitrogen) were used to visualize ZO-1 protein and F-actin. Anti-ZO-1 was diluted at 1:100 and phalloidin were diluted 1:40 according to the manufacturer’s instructions. Confocal fluorescence images were obtained on Zeiss LSM 780 microscope (National Heart, Lung and Blood Institute, Light Microscopy Core Facility). Images were analyzed by Imaris (https://imaris.oxinst.com/) and ImageJ software (https://imagej.net/ij/download/).

### Protein sample preparation and phosphopeptide enrichment for LC-MS/MS

Cells were washed with ice-cold PBS three times and lysed with 100 mM triethyl ammonium bicarbonate buffer (90114, Thermo Fisher Scientific) containing 1% SDS and protease and phosphatase inhibitors (78445, Thermo Fisher Scientific). The collected lysates were loaded onto a QIA-shredder (79656, Qiagen) and centrifuged according to the manufacturer’s instruction. Then, protein concentration was measured by BCA Protein Assay kit (23227, Thermo Fisher Scientific). Each sample (500 μg of protein) was reduced with 24 mM DTT (A39255, Thermo Fisher Scientific) for 1 h at RT and alkylated with 17 mM iodoacetamide (90034, Thermo Fisher Scientific) for 1 h at RT in dark. Pre-chilled acetone at −20 °C was added at a 6:1 ratio to the sample and incubated overnight at −20 °C to precipitate proteins. The precipitated proteins were resuspended with 100 mM triethyl ammonium bicarbonate buffer and digested with 10 μg of trypsin/LysC (V5073, Promega) at 37 °C for 18 h. The peptide concentration was measured using Pierce Quantitative Colorimetric Peptide Assays (Thermo Fisher Scientific, 23275). Five hundred micrograms of peptides from each sample were labeled using TMT11plex Isobaric Label Reagent (A37725, Lot no. VD299910, Thermo Fisher Scientific) per manufacturer’s instructions. The TMT-labeled peptides from each sample were pooled, desalted using hydrophilic-lipophilic-balanced extraction cartridges (WAT094225, Oasis), then subjected to high pH reversed phase fractionation into 24 fractions. From each fraction, 5% of the total was taken for total proteomics and the remainder was used for phospho-proteomics. Phosphopeptides were enriched sequentially using High-Select TiO_2_ and High-Select Fe-NTA phosphopeptide enrichment kits (A32993 and A32992, Thermo Fisher Scientific). Samples were vacuum dried and stored at −80 °C until LC-MS/MS analysis.

Dried peptides were resuspended with 0.1% formic acid (FA) in LC-MS grade water (J.T. Baker) before mass spectrometry analysis. Total and phospho-peptides were analyzed using a Dionex UltiMate 3000 Nano LC system connected to an Orbitrap Fusion Lumos mass spectrometer equipped with an EASY-Spray ion source (Thermo Fisher Scientific). The peptides were fractionated with a reverse-phase EASY-Spray PepMap column (C18, 75 μm × 50 cm) using a linear gradient of 4 to 32% acetonitrile (ACN) in 0.1% FA (120 min at 0.3 μl/min). The default Thermo MS2 workflow was selected on the mass spectrometer for TMT quantification.

### *In vitro* phosphorylation assay

Six synthetic peptides, each consisting of 13 amino acids as shown in Table 1, were generated. For positive and negative controls, synthetic AQP2 (QSVELHSPQSLPRGSKA), syntide2 (PLARTLSVAGLPGKK, AS-22552, AnaSpec), and CDK7tide (FLAKSFGSPNRAYKK, Dundee) were used. A mixture of synthetic peptides, each at a concentration of 0.5 nmol/ml prior to dilution, was incubated with 100 μM ATP (A50-09, Signalchem) and 1× Ca2+/Calmodulin solution II (C02-39B, Signalchem) in kinase assay buffer (K01-09, Signalchem) for 1 h at 30 °C in the absence or presence of Camk2d (02-111, Carnabio) at a concentration of 0, 0.75, and 3.75 pmol, with triplicate samples. To retrieve the peptides from the samples, samples were filtered using Amicon Ultra-0.5 Centrifugal Filter Unit (UFC503024, MilliporeSigma), followed by vacuum drying. The dried samples were reconstituted with 20 μl 0.1% FA. Pierce C18 tips were prepared by wetting them through aspirating and dispensing with 100% ACN and then equilibrated by repeating the same process with 0.1% FA. The samples were aspirated and dispensed slowly 10 to 15 times, and tips were rinsed with 10 μl 0.1% FA multiple times. Samples were then eluted by slowly aspirating and dispensing with 50 μl 50% ACN/0.1% FA into new tubes. The eluted samples were vacuum-dried and stored at −80 °C until LC-MS/MS analysis.

### LC-MS/MS data processing and analysis

For phosphoproteomics, the raw mass spectra were searched against the mouse UniProt reference proteome (UP000002494_10116.fasta, downloaded in Sep. 2021) using MaxQuant 1.6.17.0 with the default common contaminant library, and lot-specific TMT isotopic impurity correction factors were used as recommended in the TMT product data sheets. “Trypsin/P” was set as the digestion enzyme with up to two missed cleavages allowed. Carbamidomethylation of cysteine (C) was configured as a fixed modification. Variable modifications included phosphorylation of serine, threonine, and tyrosine (S, T, Y) and oxidation of methionine (M). The false discovery rate (FDR) was limited to 1% using the target-decoy algorithm. Other parameters were kept as the defaults. The raw mass spectra data and all MaxQuant search output files are deposited to the ProteomeXchange Consortium *via* the PRIDE partner repository (85, 86) with the data identifier PXD040159. Effects of Camk2d KO were quantified by Log_2_ ratios of MS2 reporter ion intensities (corrected by TMT isotopic distributions) between Camk2d KO and intact cells.

For *in vitro* phosphorylation, the raw mass spectra were searched against the synthesized peptide sequences using MaxQuant 2.4.4.0 with no enzyme digestion mode. Variable modifications included phosphorylation of serine and threonine (S, T) and oxidation of methionine (M). Quantification included both modified and unmodified peptides by unchecking the discard unmodified counterpart peptides option. The FDR was limited to 1% using the target-decoy algorithm. The “Phospho intensity” values were extracted from MaxQuant’s output “Phospho (STY)Sites.txt”. The “Total intensity” values were extracted from MaxQuant’s output “proteinGroups.txt” that indicate the sum of intensity values from both phosphorylated and unphosphorylated peptides.

### RNA isolation and sequencing

Camk2d intact and KO clones were cultured on a permeable membrane filter system for long-term dDAVP treatment (Cell culture section above). Total RNA was isolated using Direct-zol RNA Microprep kit (R2062, Zymo Research) following the manufacturer’s instructions. First-strand cDNA was prepared from 50 ng of total RNA using SMART-Seq v4 Ultra Low Input RNA Kit, followed by cDNA amplification. After purification of amplified cDNA using the AMPure XP beads (A63880, Beckman Coulter), the quantity of the synthesized cDNA was examined using the Qubit dsDNA HS DNA assay kit (Q32851, Invitrogen). One nanogram of the synthesized cDNAs were fragmented and tagged with index primers (FC-131-1024, Nextera XT DNA library Preparation Kit, Illumina) following the manufacturer’s protocol. RNA-seq was performed by 50-bp paired-end NovaSeq (Illumina). Ten samples were loaded in each lane. Raw sequencing reads were aligned by STAR 2.7.10 b to the mouse reference genome (Ensembl genome 106). Transcript per million and expected counts were generated by RSEM 1.3.1. Expected counts from in Camk2d KO (n = 5) and control (n = 5) samples were used for differential gene expression analysis using edgeR 3.38.4.

### Bioinformatics and statistics

Most analyses were carried out using Microsoft Excel (https://www.microsoft.com/en-us/microsoft-365/excel) and R software (https://www.r-project.org/). MaxQuant’s output “proteinGroups.txt” was used in total proteome analysis. Eleven thousand five hundred seventy monophosphosite information were extracted from MaxQuant’s output “Phospho (STY)Sites.txt” and further used for the following Camk2d substrate, motif, and gene ontology analyses. Statistical calculations used base-2 log transformation. *p*_joint_ < 0.0005 was used to identify significantly changed total proteins or phosphosites. For the identification of putative direct targets of Camk2d, phosphosites with R/K in position −3 and D/E in position +2 were further filtered with dual criteria (*p*_joint_ < 0.0005 and |Log_2_(KO/Ctrl)| > 0.3). *p*_joint_ is for a given protein, phospho-site is the product of the *p* value based on the t-statistic and the area under the Gaussian distribution curve from |Z| to positive infinity where Z is the log_2_(KO/Control) divided by the SD across all log_2_(KO/Control) values. Motif preferences were determined using *PTMLogo* (33). The inputs were 13 centralized amino acid sequences around the phosphorylation site (S/T). The preferred motifs were identified as motifs with Chi-square < 0.001. The Database for Annotation, Visualization and Integrated Discovery (DAVID) (https://david.ncifcrf.gov/, RRID:SCR_001881) was used to identify gene set enrichment on the gene ontology database. The enrichment of specific gene ontology biological processes was defined by statistical evaluation using Fisher Exact test (*p* value < 0.05).

## Data availability

These data have been made available to users on a publicly accessible webpage allowing searching, browsing, and download the results (https://esbl.nhlbi.nih.gov/Databases/CAMK2D-proteome/). The raw proteomics data are accessible to users at the ProteomeXchange Consortium *via* the PRIDE database (https://www.ebi.ac.uk/pride/, PXD040159). RNA-seq data is deposited in Gene Expression Omnibus (GEO) (https://www.ncbi.nlm.nih.gov/geo/query/acc.cgi?acc=GSE228762.

## Supporting information

This article contains Supporting information.

## Conflict of interest

The authors declare that they have no conflicts of interest with the contents of this article.
