# Supplementary material for: Using CRISPR-Cas9/phosphoproteomics to identify substrates of calcium/calmodulin-dependent kinase 2δ
Source: J Biol Chem. 2023 Oct 20;299(12):105371. doi: 10.1016/j.jbc.2023.105371 (PMC10783575; doi:10.1016/j.jbc.2023.105371)
Supplement: Supplemental Table S1 [file mmc2.docx]

**Supplemental Table 1. CRISPR/Cas9-mediated genomic mutations of Camk2d**

| **KO**  **Clones** | **Target**  **Exon** | **Mutations** | | | |
| --- | --- | --- | --- | --- | --- |
| KO 1 | Exon 8 | 2-bp ins | 1-bp del |  |  |
| KO 2 | Exon 8 | 1-bp ins | 11-bp del |  |  |
| KO 3 | Exon 9 | 1-bp del | 24-bp del |  |  |
| KO 4 | Exon 9 | 2-bp del | 66-bp del | 74-bp del | 2-bp ins, 77-bp del |
| KO 5 | Exon 9 | 1-bp del | 4-bp del | 41-bp del |  |

del, deletion; ins, insertion
